# Ultrasensitive PSA: rethinking post-surgical management for node positive prostate cancer

**DOI:** 10.3389/fonc.2024.1363009

**Published:** 2024-04-09

**Authors:** Jonathan A. Aguiar, Eric V. Li, Austin Ho, Richard Bennett, Yutai Li, Clayton Neill, Edward M. Schaeffer, Hiten D. Patel, Ashley E. Ross

**Affiliations:** Feinberg School of Medicine, Department of Urology, Northwestern University, Chicago, IL, United States

**Keywords:** prostate cancer, lymph node metastases, ultrasensitive PSA, node positive prostate cancer, post-surgical management

## Abstract

**Introduction:**

Clinicians may offer patients with positive lymph nodes (pN1) and undetectable PSA following surgery for prostate cancer either observation or adjuvant therapy based on AUA, EAU, and NCCN guidelines considering standard PSA detection thresholds of <0.1ng/ml. Here we sought to investigate the outcomes of pN1 patients in the era of ultrasensitive PSA testing.

**Methods:**

We queried the Northwestern Electronic Data Warehouse for patients with prostate cancer who were pN1 at radical prostatectomy and followed with ultrasensitive PSA. Patients receiving neoadjuvant treatment were excluded. We compared clinical characteristics including age, race, pre-operative PSA, Gleason grade, tumor stage, surgical margins, and nodal specimens to identify factors associated with achievement and maintenance of an undetectable PSA (defined as <0.01 ng/mL). Statistics were performed using t-test, Mann-Whitney U test, chi-squared analysis, and logistic regression with significance defined as p<0.05.

**Results:**

From 2018-2023, 188 patients were included. Subsequently, 39 (20.7%) had a PSA decline to undetectable levels (<0.01 ng/mL) post-operatively at a median time of 63 days. Seven percent of these men (3/39) were treated with adjuvant RT + ADT with undetectable PSA levels. 13/39 (33.3%) had eventual rises in PSA to ≥0.01 ng/mL for which they underwent salvage RT with ADT. Overall, 23/39 (59%) patients achieved and maintained undetectable PSA levels without subsequent therapy at median follow-up of 24.2 mo. Compared to patients with PSA persistence after surgery or elevations to detectable levels (≥0.01 ng/mL), patients who achieved and maintained undetectable levels had lower Gleason grades (p=0.03), lower tumor stage (p<0.001), fewer positive margins (p=0.02), and fewer involved lymph nodes (p=0.02). On multivariable analysis, only primary tumor (pT) stage was associated with achieving and maintaining an undetectable PSA; pT3b disease was associated with a 6.6-fold increased chance of developing a detectable PSA (p=0.03).

**Conclusion:**

Ultrasensitive PSA can aid initiation of early salvage therapy for lymph node positive patients after radical prostatectomy while avoiding overtreatment in a significant subset. 20% of patients achieved an undetectable PSA and over half of this subset remained undetectable after 2 years.

## Introduction

With an estimated 1.4 million new cases diagnosed in 2020, prostate cancer remains a significant global health burden as the second most diagnosed cancer and the fifth leading cause of cancer death among men worldwide (1, 2). Along with significant advancements, the management of prostate cancer continues to evolve and move towards more personalized treatment strategies.

The Prostate-Specific Antigen (PSA) test, since its approval by the FDA in 1986, has been a cornerstone in the detection and monitoring of prostate cancer recurrence post-surgery. After radical prostatectomy, a rise in PSA levels is often the first indicator of recurrent disease, even before radiographic evidence or symptoms become apparent (3). The American Urological Association (AUA) and the European Association of Urology (EAU) recommend regular PSA testing post-surgery as a surveillance strategy to identify biochemical recurrence, historically defined as a PSA level of 0.2 ng/mL or higher, following radical prostatectomy. In contemporary practice, PSA assays have advanced such that it is now routinely possible to detect serum levels as low as 0.001 ng/mL (4, 5).

Additionally, the prognosis of lymph node involvement following pelvic lymph node dissection (PLND) at the time of radical prostatectomy (pN1) has been well studied and these patients have heterogenous cancer specific survival (6). It is estimated that 12-15% of men will be positive for lymph node metastases at the time of their surgery (7, 8). Among node positive patients, previous work has also demonstrated that persistently elevated post-operative PSA’s (>0.2 ng/mL) are associated with poorer metastases-free survival (9, 10). Current AUA and EAU guidelines state that men with pN1 disease and undetectable post-operative serum PSA levels can be offered either adjuvant therapy or observation (11). Although early adjuvant androgen deprivation therapy (ADT) with or without radiotherapy (RT) for these patients has been shown to improve biochemical control and other oncologic outcomes it has treatment-related toxicity and in some cases may represent overtreatment (12–15). Current AUA, EAU, and NCCN recommendations on offering either adjuvant treatment or surveillance are primarily based on standard PSA detection thresholds of >0.1 ng/mL (16, 17). Neither guideline body provides strong evidence for diagnostic or therapeutic plans for men with pN1 and undetectable ultra-sensitive PSA’s.

Given this paucity of research in the era of ultrasensitive assays, we seek to investigate clinical outcomes and PSA trajectories of pN1 men at our institution followed with ultrasensitive PSA. Furthermore, we seek to identify which pN1 patients are most likely to achieve and maintain undetectable PSA levels post-surgery and its subsequent implications for planning adjuvant or salvage treatment strategies.

## Materials and methods

### Data acquisition

The Northwestern Electronic Data Warehouse was queried for patients seen within the Northwestern Medical Group who had prostate cancer demonstrating pathologic lymph node involvement (pN1) at the time of radical prostatectomy from January 1, 2018, to October 20, 2023. Northwestern Medical Group is comprised of 11 hospitals and associated clinics located throughout the state of Illinois. Patient data utilized in this study was approved by the Northwestern University Institutional Review Board in accordance with the IRB #STU00213284. A total of 202 (5.2%) of 3835 patients who underwent radical prostatectomy within the Northwestern Medical Group network were pN1 during the specified timeline. Patients were excluded from final analyses if they had received neoadjuvant therapy (n=12) or if they were subsequently lost to post-operative follow-up (n=2). 188 patients were included in our final analyses.

### Patient cohort clinical characteristics

The following clinical and demographic characteristics were collected: age at time of surgery, baseline serum pre-operative PSA, Charlson Comorbidity Index (CCI), self-identified ethnicity, self-identified race, Gleason Grade at surgical pathology, pathologic primary tumor stage, surgical margin involvement, and lymph node involvement. All subsequent post-operative ultrasensitive serum PSA values were collected to analyze individual patient trends. In our study we defined undetectable PSA as a serum value <0.01 ng/mL. Associated surgical pathology reports and pre-operative staging imaging reports (including prostate MRI, CT abdomen and pelvis, PSMA PET/CT scans, Fluciclovine F-18 scans, and bone scans) were also collected. Age, CCI, PSA, and lymph node involvement (total nodes resected, total positive nodes, and lymph node density) were assessed as continuous variables. Categorical variables included ethnicity (Hispanic/Latino, Not Hispanic/Latino), race (White, Black or African American, Asian, Declined or Other), Gleason Grade, pathologic primary tumor stage, and surgical margin involvement (any positive margins, unifocal/multifocal margin involvement, and linear involvement ≥3mm).

### Statistical analyses

Statistical analyses were performed using Stata/SE (Version 17.0). Mann-Whitney U test was used to compare medians of pre-operative PSA. Categorical variables were analyzed utilizing chi-square tests. The remaining continuous variables were analyzed with unpaired t-tests. Statistical significance was defined as p<0.05.

Univariable and multivariable logistic regressions were performed to identify factors associated with achieving and maintaining an undetectable serum PSA with no further adjuvant or salvage therapy. The variables of interest for the univariable models included age, baseline PSA, CCI, ethnicity, race, Gleason Grade, pathologic tumor stage, surgical margin involvement, and lymph node involvement. Individual components found to be statistically significant under univariable regression were subsequently analyzed in a multivariable regression.

## Results

### Cohort characteristics and PSA trends

Between January 2018 and October 2023, 3835 radical prostatectomies were performed within the Northwestern Medical Group. 202 (5.2%) patients were found to be pN1. Twelve individuals received neoadjuvant treatment and two were lost to follow-up postoperatively. Therefore, a total of 188 men at our institution who underwent radical prostatectomy and were found to have pathologic lymph node involvement were followed with ultrasensitive PSA assays. 16 (8.5%) of these patients were clinically node positive (cN1) prior to undergoing surgery based on primary staging. Nine individuals were cN1 based on PSMA PET/CT scan and seven staged through conventional imaging, specifically multiparametric prostate MRI. At final pathology, most patients had high grade and high stage disease with 52.5% diagnosed with Gleason Grade group 5 disease and 53.7% with T3b pathologic tumor stage. Although a significant subset, 14 (7.4%) patients, presented with Gleason Grade group 2 disease. Similarly, 13 (6.9%) patients were staged at pT2 on final pathology. A median number of 12 (IQR 8 – 17) nodes were resected operatively during the pelvic lymph node dissection with a median positive lymph node density of 0.142 (IQR 0.076 – 0.250). PSA persistence (serum PSA levels that remained ≥0.01 ng/mL) was observed in 129 patients (79.3%).

**Figure 1:**
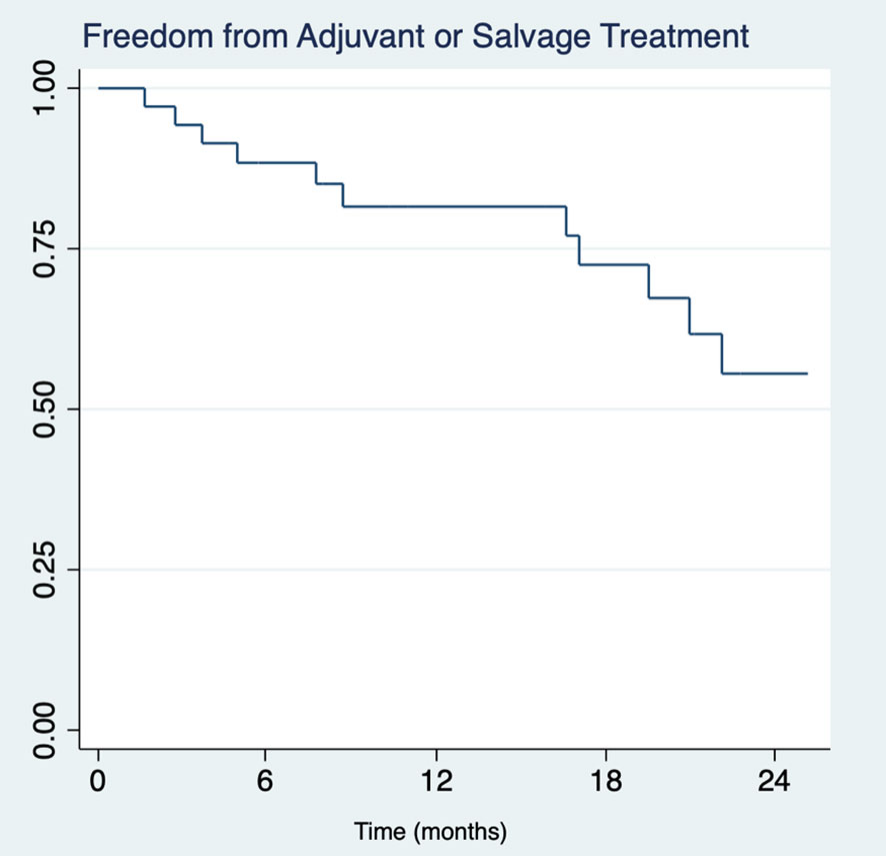
Kaplan-Meier survival analysis of freedom from adjuvant or salvage therapy in pN1 patients (n=39) with undetectable serum PSA (<0.01 ng/mL) across two years.

Post-operative PSA tracking revealed that 39 patients (20.7%) achieved and maintained undetectable serum PSA levels (<0.01 ng/mL), within a median timeframe of 63 days (IQR 51 – 110). Upon subsequent follow-up, 13 of these patients (33.3%) experienced a rise in PSA levels to detectable ≥0.01 ng/mL levels and underwent salvage radiation therapy (RT) with androgen deprivation therapy (ADT) (**Figure 1**). Median interval from completion of surgery to initiation of salvage therapy was 385 days (IQR 14 – 605). Notably, a small subset of 3 individuals (7.7%) with undetectable PSA levels were treated with true adjuvant RT + ADT. Of the remaining patients who achieved undetectable PSA levels, 23 (59.0%) underwent no further treatment within our cohort’s median follow-up interval of 24.2 months.

### Factors associated with achieving and maintaining an undetectable PSA

Patients that sustained undetectable post-operative serum PSA levels exhibited significantly favorable clinical profiles, including lower Gleason grades (p=0.03), less advanced primary tumors stages (p<0.001), lower positive surgical margin rates (p=0.02), and lower number of positive lymph nodes (p<0.02) compared to their counterparts with PSA persistence or recurrence (**Table 1**). There were no statistically significant differences between both groups with respect to age, baseline PSA, clinical node positivity status (cN1), CCI, ethnicity, or race. On multivariable logistic regression, patients with pT3b disease had lower likelihood of achieving and maintaining an undetectable PSA (OR = 0.15, 95% CI: 0.02 – 0.89, p=0.03) (**Table 2**).

**Table 1:** Characteristics of patients lymph node positive at radical prostatectomy (pN1).

	PSA Persistence, Recurrence, or Further Treatment	Undetectable and No Further Treatment	p-value
	n = 165 [95%CI]	n = 23 [95%CI]	
**Age (years)**	63.9 [62.8 - 64.9]	66.2 [62.7 - 69.6]	0.15
**Pre-Operative PSA (ng/mL)^1^**	8.78 [5.28 - 20.48]	6.13 [4.93 - 10.68]	0.21
**CCI**	6.3 [5.9 - 6.7]	6.1 [5.1 - 7.1]	0.22
**Hispanic/Latino Ethnicity**	10 (6.1%)	0 (0%)	0.23
Self-Identified Race			0.71
White	115 (69.7%)	19 (70.3%)	
Black or African American	15 (9.1%)	2 (8.7%)	
Asian	7 (4.2%)	0 (0%)	
Declined/Other	28 (17.0%)	3 (13.0%)	
**Clinically Node Positive (cN1)**	13 (7.8%)	3 (13.0%)	0.4
Gleason Grade			0.03
GG 2	10 (6.1%)	4 (17.4%)	
GG 3	51 (30.9%)	11 (47.8%)	
GG 4	12 (7.3%)	2 (8.7%)	
GG 5	92 (55.7%)	6 (26.1%)	
Primary Tumor Stage			<0.001
pT2	5 (3.0%)	8 (34.8%)	
pT3a	65 (39.4%)	9 (39.1%)	
pT3b	95 (57.6%)	6 (26.1%)	
Surgical Margins			
Positive Margin	105 (63.6%)	9 (39.1%)	0.02
Unifocal Involvement	38 (23.0%)	4 (17.4%)	0.54
Multifocal Involvement	67 (40.6%)	5 (21.7%)	0.08
Linear Margin Involvement ≥ 3mm	49 (29.7%)	2 (8.7%)	0.01
Lymph Nodes			
Total Lymph Nodes Resected	13.3 [12.2 - 14.4]	12.2 [9.5 - 14.9]	0.46
Number of Positive Lymph Nodes	2.3 [1.9 - 2.7]	1.3 [1.0 - 1.6]	0.02
Lymph Node Density	0.19 [0.17 - 0.22]	0.14 [0.10 - 0.19]	0.07

^1^Median [IQR].

**Table 2:** Multivariable logistic regression for achievement and maintenance of undetectable PSA with no further therapy.

	OR [95% CI]	p-value
Gleason Grade		
GG 2	–	–
GG 3	0.88 [0.16 - 4.75]	0.89
GG 4	0.37 [0.02 - 5.19]	0.46
GG 5	0.34 [0.06 - 1.92]	0.22
Primary Tumor Stage		
pT2	–	–
pT3a	0.28 [0.06 - 1.23]	0.09
pT3b	0.15 [0.02 - 0.89]	0.03
**Any Positive Surgical Margins**	1.72 [0.34 - 8.50]	0.5
**Linear Margin Involvement** ≥ **3mm**	0.24 [0.03 - 1.73]	0.16
**Number of Positive Lymph Nodes**	0.88 [0.23 - 10.56]	0.64

Model excludes the three individuals treated with immediate adjuvant therapy

As a secondary endpoint, we sought to assess variables associated with achieving an undetectable PSA post-operatively versus PSA persistence (≥0.01 ng/mL). Similarly, patients that achieved undetectable serum PSA levels post-operatively exhibited lower Gleason grades (p=0.02), less advanced primary tumor stages (p<0.001), lower positive surgical margin rates (p<0.001), fewer positive lymph nodes (p=0.008), and lower lymph node densities (p<0.02) (**Supplementary Table 1**). On multivariable logistic regression, none of the aforementioned variables demonstrated statistically significant associations with achieving undetectable serum PSA levels (**Supplementary Table 2**).

## Discussion

Our study investigates the outcomes and PSA trajectories of patients with node-positive (pN1) prostate cancer in the context of ultrasensitive PSA assays. Our findings show that a significant proportion of men (20.7%) achieved undetectable ultrasensitive PSA levels post-radical prostatectomy, with nearly 60% of this subset maintaining these levels without need for further intervention over approximately two years of follow-up. Our study investigates the outcomes and PSA trajectories of patients with node-positive (pN1) prostate cancer in the context of ultrasensitive PSA assays. Our findings show that a significant proportion of men (20.7%) achieved undetectable ultrasensitive PSA levels post-radical prostatectomy, with nearly 60% of this subset maintaining these levels without need for further intervention over approximately two years of follow-up. This presents an opportunity to revisit current surveillance and treatment paradigms for these select patients. More specifically, we believe these data provide further validation on surveillance as a viable management option for pN1.

Current guidelines suggest the option of adjuvant therapy or observation for patients with pN1 disease following surgery. However, these recommendations, which are predominantly based on standard PSA detection thresholds, may not fully account for the nuanced understanding afforded by ultrasensitive assays. Our data indicate that ultrasensitive PSAs could aid providers in making more individualized decisions regarding post operative management for pN1 post-prostatectomy patients.

In our cohort, lower Gleason grades, less advanced tumor stages, and lower positive surgical margin rates were associated with the achievement and maintenance of undetectable PSA levels. Notably, patients with pT3b disease were significantly less likely to achieve and maintain undetectable PSA levels. These findings corroborate the prognostic value of primary tumor staging in predicting recurrence and align with previous studies highlighting the importance of local tumor stage in adverse postoperative outcomes especially among pN1 patients (18). While it is widely recognized that patients with unfavorable characteristics are more apt to benefit from adjuvant therapies, our analysis revealed that a substantial segment of patients, despite possessing such adverse factors as elevated Gleason scores and seminal vesicle invasion, successfully achieved and maintained undetectable levels of ultrasensitive PSA. This finding suggests that ongoing monitoring with ultrasensitive PSA could offer a practical surveillance strategy for these individuals, potentially minimizing unnecessary treatments. Moreover, the use of ultrasensitive PSA facilitates the initiation of salvage treatments at earlier stages compared to conventional PSA thresholds, ensuring that patients receive prompt and appropriate intervention when needed.

Overall, retrospective studies have suggested that a significant number of pN1 patients may experience long-term, disease-free survival even in the absence of subsequent therapies. For example, one retrospective series found that nearly a third of patients managed with initial observation would be free from biochemical recurrence (defined as >0.2 ng/mL) and 65% free of distant metastases even at 10 years of follow up (19). Additionally, consistent with our series, pN1 patients are heterogenous in nature; various adverse pathologic features significantly impact oncologic control (i.e., higher tumor grades, surgical margin positivity, seminal vesicle invasion). Patients with these adverse features are more likely to require local control irrespective of lymph node involvement. Additionally, lymph node density has also been shown to shown to influence recurrence free survival with patients demonstrating 1-2 positive nodes having a clinical recurrence free survival of 70-73% compared to 49% in those with at least 5 involved nodes at 10 years based on measurable imaging findings (20).

Within the AUA guideline statement on post-surgical pN1 patients, recommendations surrounding adjuvant treatment versus observation rely heavily on a prospective randomized trial, ECOG 3886, that demonstrated that immediate adjuvant ADT was associated with improved PFS (HR=3.42, 95% CI: 1.96 - 5.98), prostate cancer specific survival (HR=4.09, 95% CI: 1.76 – 9.49), and overall survival (HR = 1.84, 95% CI: 1.01 – 3.35) at a median follow-up of 11.9 years when compared to delayed ADT (11, 13). However, this specific trial did not compare immediate adjuvant ADT versus ADT initiated at the detection biochemical recurrence. Because the trial was conducted prior to modern PSA screening, ADT in the delayed treatment arm was with withheld until the detection of distant metastases. For these reasons, we believe this trial holds poor generalizability to the current prostate cancer treatment landscape.

To our knowledge, the only data analyzing the role of post-operative adjuvant or salvage radiation for pN1 men is observational and/or retrospective in nature. Several studies conducted have demonstrated improvements in survival when adjuvant RT is combined with ADT (21–25). For example, a National Cancer Database study suggested improved survival for RT + ADT over ADT alone or observation in the overall cohort, but it appeared a subset of 32% of patients without adverse features derived no benefit form adjuvant therapy (26). However, none of these cohort studies have directly compared use of adjuvant RT + ADT to surveillance with early salvage based on PSA monitoring. Additionally, there are only two retrospective studies to our knowledge which, in pN1 men, compared RT + ADT to observation followed by salvage treatment in the setting of biochemical recurrence. In the first cohort, the authors found that RT + ADT was associated with better overall survival (HR=0.41, 95% CI: 0.27 – 0.64, p<0.001) and cancer specific survival (HR=0.64, 95% CI: 0.43 – 0.95, p=0.027) (16). The authors heterogeneously defined biochemical recurrence by the three institutional sites included in the study: ≥0.1 ng/mL, ≥0.2 ng/mL, and ≥0.4 ng/ml. Similarly, the second study also defined biochemical recurrence and eligibility for salvage therapy at a PSA threshold of ≥0.2 ng/mL (27).

Our data should also be considered within the context of recent prospective data. Specifically, the STAMPEDE trial has demonstrated long-term, improved metastases-free survival with ADT and androgen receptor-targeted agents in high-risk/N1 patients. Consequently, after publication of these data, the role of surgery in the management of N1 prostate cancer should thus be ideally considered within prospective trials or following in-depth discussion with patients. This approach ensures that treatment strategies for N1 patients are both evidence-based and tailored to individual patient needs.

Additionally, recent advancements in prostate cancer imaging have provided additional tools in the post-operative evaluation of suspected recurrence. Prostate Specific Membrane Antigen (PSMA) based imaging is the preferred modality to assess patients for cancer recurrence or persistence following radical prostatectomy due to its high sensitivity compared to traditional imaging modalities such as multiparametric MRI and CT (28). Traditional cross-sectional imaging is highly dependent on subtle anatomic changes and has demonstrated especially poor performance in patients with low serum PSA values (<2.0 ng/mL) (29, 30). For this reason, PSMA PET/CT has allowed for higher detection rates of metastatic disease particularly in the lower PSA range (<0.2 ng/mL) (31). In a separate institutional study where we assessed factors associated with PSMA positivity at low serum PSA levels post-surgery, we found that although individuals with higher PSA levels (i.e. ≥1 ng/mL) had the highest probability of demonstrating PSMA-positive disease, nearly 40% of patients with serum PSA <0.2 ng/mL had positive imaging concerning for recurrence. The precision of PSMA PET/CT in detecting prostate cancer recurrence at lower PSA levels underscores its potential to significantly inform treatment decisions, particularly in the context of ultrasensitive PSA monitoring. This earlier detection may facilitate prompt intervention and reduce delay in time to salvage treatments which demonstrate their highest efficacy when tumor burden is minimal.

Limitations of our study include its retrospective design and the relatively short median follow-up period of 24.2 months for patients in our cohort. We acknowledge that approximately 2 years of follow-up is helpful but insufficient in assessing PSA durability and effects on longer-term oncologic outcomes. Further research with extended follow-up is necessary to validate these findings. Additionally, we acknowledge there is a selection bias from our high-volume prostate cancer center that may not be representative of other practices. Additionally, genomic testing with tests like Decipher might serve as supplementary prognostic indicators for PSA recurrence in patients with positive lymph nodes at prostatectomy. In our series, however, only 4 men had completed Decipher assays at the time of our data query and thus this was not evaluated in our analyses.

In summary, 20% of pathologically lymph node positive patients achieved an undetectable PSA with most sustaining disease remission after surgery alone and others receiving prompt early salvage therapy. Our data, though limited by short follow-up, suggest that pN1 patients could consider observation with ultrasensitive PSAs as opposed to immediate adjuvant therapy, particularly if they lack other adverse features such as seminal vesicle involvement and high Gleason Grade. Such a strategy could reduce over-treatment of prostate cancer.

## Data availability statement

The datasets presented in this article are available upon request to protect patient confidentiality. Readers interested in additional information regarding the study’s data please contact the corresponding author.

## Ethics statement

The studies involving humans were approved by Northwestern University Institutional Review Board. The studies were conducted in accordance with the local legislation and institutional requirements. Written informed consent for participation was not required from the participants or the participants’ legal guardians/next of kin because of the nature involving retrospective review of existing patient data.

## Author contributions

JA: Writing – original draft, Writing – review & editing. EL: Writing – original draft, Writing – review & editing. AH: Writing – original draft, Writing – review & editing. RB: Writing – original draft, Writing – review & editing. YL: Writing – original draft, Writing – review & editing. CN: Writing – original draft, Writing – review & editing. ES: Writing – original draft, Writing – review & editing. HP: Writing – original draft, Writing – review & editing. AR: Writing – original draft, Writing – review & editing.

## Supplementary material

Supplementary Table 1 Characteristics of patients with PSA persistence post-surgery versus patients that achieved undetectable PSA.

Supplementary Table 2 Multivariable logistic regression for achievement of undetectable post-operative PSA.
