# Supplementary material for: Ultrasensitive PSA: rethinking post-surgical management for node positive prostate cancer
Source: Front Oncol. 2024 Apr 9;14:1363009. doi: 10.3389/fonc.2024.1363009 (PMC11035792; doi:10.3389/fonc.2024.1363009)
Supplement: Supplementary Table 1 — Characteristics of patients with PSA persistence post-surgery versus patients that achieved undetectable PSA. [file Table_1.docx]

Supplementary Table 1: Characteristics of patients with PSA persistence post-surgery versus patients that achieved undetectable PSA.

|  | **PSA Persistence** | **Achieved Undetectable PSA** |  |
| --- | --- | --- | --- |
|  | n = 149 [95%CI] | n = 39 [95%CI] | p-value |
| **Age (years)** | 63.7 [62.6 - 64.9] | 65.4 [63.2 - 67.7] | 0.19 |
| **Pre-Operative PSA (ng/mL)^1^** | 9.02 [5.44 – 20.97] | 6.17 [4.91 – 10.35] | 0.05 |
| **CCI** | 6.3 [5.9 - 6.7] | 6.0 [5.1 - 6.8] | 0.51 |
| **Hispanic/Latino Ethnicity** | 9 (6.0%) | 1 (2.5%) | 0.39 |
| **Self-Identified Race** |  |  | 0.83 |
| White | 104 (69.8%) | 29 (74.3%) |  |
| Black or African American | 14 (9.4%) | 3 (7.6%) |  |
| Asian | 5 (3.3%) | 2 (5.1%) |  |
| Declined/Other | 26 (17.4%) | 5 (12.8%) |  |
| **Clinically Node Positive (cN1)** | 13 (8.7%) | 3 (7.6%) | 0.8 |
| **Gleason Grade** |  |  | 0.02 |
| GG 2 | 9 (6.0%) | 5 (12.8%) |  |
| GG 3 | 47 (31.5%) | 15 (38.4%) |  |
| GG 4 | 8 (5.3%) | 6 (15.3%) |  |
| GG 5 | 85 (57.0%) | 13 (33.3%) |  |
| **Primary Tumor Stage** |  |  | <0.001 |
| pT2 | 4 (2.6%) | 9 (23.0%) |  |
| pT3a | 55 (36.9%) | 19 (48.7%) |  |
| pT3b | 90 (60.4%) | 11 (28.2%) |  |
| **Surgical Margins** |  |  |  |
| Positive Margin | 101 (67.7%) | 13 (33.3%) | <0.001 |
| Unifocal Involvement | 37 (24.8%) | 5 (12.8%) | 0.11 |
| Multifocal Involvement | 64 (42.9%) | 8 (20.5%) | 0.01 |
| Linear Margin Involvement ≥ 3mm | 47 (43.5%) | 4 (12.1%) | <0.001 |
| **Lymph Nodes** |  |  |  |
| Total Lymph Nodes Resected | 13.4 [12.2 - 14.5] | 12.1 [10.2 - 14.1] | 0.31 |
| Number of Positive Lymph Nodes | 2.4 [2.0 - 2.8] | 1.3 [1.1 - 1.5] | 0.008 |
| Lymph Node Density | 0.20 [0.17 - 0.22] | 0.13 [0.11 - 0.16] | 0.02 |

1: Median [IQR]

Supplementary Table 2: Multivariable logistic regression for achievement of undetectable post-operative PSA.

|  | **OR [95% CI]** | **p-value** |
| --- | --- | --- |
| **Gleason Grade** |  |  |
| GG 2 | -- | -- |
| GG 3 | 0.68 [0.13 - 3.39] | 0.64 |
| GG 4 | 2.51 [0.33 - 18.91] | 0.37 |
| GG 5 | 0.46 [0.09 - 2.27] | 0.34 |
| **Primary Tumor Stage** |  |  |
| pT2 | -- | -- |
| pT3a | 0.47 [0.11 - 2.04] | 0.31 |
| pT3b | 0.27 [0.05 - 1.41] | 0.12 |
| **Any Positive Surgical Margins** | 0.68 [0.15 - 2.99] | 0.61 |
| **Multifocal Margin Involvement** | Omitted due to collinearity |  |
| **Linear Margin Involvement** ≥ **3mm** | 0.40 [0.07 - 2.19] | 0.29 |
| **Lymph Node Density** | 0.50 [0.01 - 37.46] | 0.75 |
| **Number of Positive Lymph Nodes** | 0.77 [0.44 - 1.33] | 0.35 |
